# Four Decades of Foot and Ankle Research Activity: A Scientometric study of Subspecialty Foot and Ankle Journals

**DOI:** 10.12669/pjms.39.4.7229

**Published:** 2023

**Authors:** Yaohong Wu, Qin Chen, Rongchun Chen, Qi Luo

**Affiliations:** 1 Yaohong Wu, Department of Spine Surgery, Ganzhou People’s Hospital, Ganzhou, China; 2 Qin Chen, Department of Spine Surgery, Ganzhou People’s Hospital, Ganzhou, China; 3 Rongchun Chen, Department of Spine Surgery, Ganzhou People’s Hospital, Ganzhou, China; 4 Qi Luo, Department of Spine Surgery, Ganzhou People’s Hospital, Ganzhou, China

**Keywords:** Foot, Ankle, Publication, Scientometric analysis

## Abstract

**Objectives:**

To evaluate foot and ankle documents using scientometric methods and provide insight into global research activities.

**Methods:**

This scientometric study was conducted at the Department of Spine Surgery, Ganzhou People’s Hospital, China. Documents on foot and ankle from 1980 to 2019 were retrieved from the Scopus database. The number of documents, year of publication, journal, country, institution, author, h-index, and top-cited documents were analyzed.

**Results:**

In total, 11313 documents were retrieved. The annual research output on foot and ankle showed a dramatic increase over the past four decades, especially in the past decade (p = 0.000). Foot & Ankle International published the biggest number of documents (44.48%). The United States contributed more than half (52.17%) of the global production, followed by the United Kingdom (11.39%), and Germany (3.62%). The United States had the highest h-index (109). The Hospital for Special Surgery (1.87%) ranked first in terms of productivity, followed by Union Memorial Hospital (1.37%), and Duke University Medical Center (1.24%). The most productive author was Myerson MS (1.25%), followed by Schon LC (0.77%), and Hyer CF (0.74%).

**Conclusion:**

Research on foot and ankle has thrived rapidly over the past four decades, particularly in the last decade. The United States contributes the most to the quantity and quality of foot and ankle documents.

## INTRODUCTION

Foot and ankle surgery has become a critical subspecialty in the field of orthopedics. This might be attributed to the rapid progress of foot and ankle research.^1^-^8^ In light of the impact of foot and ankle research and in light of differences in medical policy and health services, the need to evaluate foot and ankle research has become crucial.^9^-^15^ Publication is an important index of the conception of new knowledge, and is also an important method for scientific communication.^3^-^8^,^16^-^18^ Scientometrics involve metrological approaches for the quantitative and qualitative analysis of scientific documents.^4^,^7^ Scientometric study is a vital element in establishing baseline data for future comparisons in certain fileds.^4^,^7^,^8^

Scientometric analysis can provide information such as publication trends and the characteristics of documents, counties, institutions, and authors.^4^-^6^ It helps understand their different contributions, and can be used to identify research gaps that future efforts could focus on.^3^,^5^,^6^,^19^ Scientometric analysis of research activity is widely used to determine the characteristics of documents on various topics.^3^-^8^ However, scientometric studies on foot and ankle publications are limited. Therefore, the goal of the current study was to perform a comprehensive analysis of publications on foot and ankle research using a scientometric method during the period 1980-2019.

## METHODS

This scientometric study was conducted at the Department of Spine Surgery, Ganzhou People’s Hospital, China. This study does not involve animals or humans, and ethical approval was not required. The method and indicators chosen in the current study have been explained in detail in similar scientometric studies.^3^-^8^ To achieve the goal of the study, we also used the Scopus database to identify documents on the foot and ankle.^3^-^8^ Scopus is one of the largest electronic databases for literature research and scientometric analysis.^8^ It has the ability to provide details on journals, citations, countries, institutions, authors, h-index, and other information based on search results.^4^-^6^

Another advantage is that it has a higher number of journals than PubMed and Web of Science.^20^ Scopus contains information for documents published in more than 21,500 titles from more than 5000 international publishers, including Medline.^20^ In addition, Scopus has multiple analytic functions that facilitate scientometric investigations of retrieved documents.^4^-^6^,^8^ Therefore, Scopus has been used in numerous previous publications in diverse fields.^3^-^8^

Five international subspecialty journals on foot and ankle, listed in the Journal Citation Reports, were included as literature sources, which were.^8^,^21^ These journals included Foot & Ankle International, Journal of Foot Ankle Research, Foot and Ankle Surgery, Foot and Ankle Clinics, and Journal of Foot & Ankle Surgery. The International Standard Serial Number (ISSN) for these journals were 1071-1007, 1944-7876, 1083-7515, 1558-1934, 1268-7731, 1460-9584, 1067-2516, 1542-2224, and 1757-1146. These ISSN were used as search terms. The search strategy included data published between 1980 and December 31, 2019. A comprehensive online search was performed using Scopus on January 03, 2022. Publication type was limited to article or review.^8^

Information on the number of documents, journals, countries, institutions, year of publication, h-index, and top-cited documents was collected and analyzed. The h-index was used to determine the document quality. A higher h-index suggests that documents had a greater influence. Scopus provided an h-index value for any set of documents. The h-index is an indicator of the importance of the authors in a particular field. However, the use of the h-index has been extended to evaluate the research activity and citation importance of a country or institution.^22^ A country, institution, or author with an h-index of n indicates that the country, institution, or author has a minimum of n documents receiving at least n citations.^8^,^22^

In addition, in terms of research activity, the 10 most productive countries, institutions, authors, and the 10 most cited documents were analyzed. Regression analysis was performed to determine the significance of the trends between 1980 and 2019. Statistical analysis was performed using SPSS version 22.0 (SPSS Inc., Chicago, IL, USA). Statistical significance was set at p<0.05.

## RESULTS

**Fig.1:**
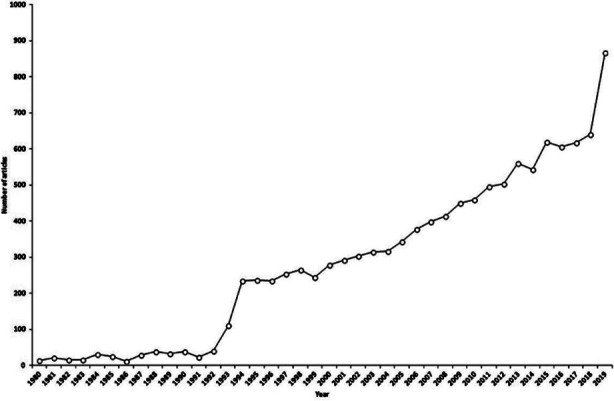
Publication trend on foot and ankle research between 1980 and 2019.

Information on the included journals is presented in Table-I. Foot & Ankle International had the highest impact factor, followed by Journal of Foot Ankle Research, and Foot and Ankle Surgery. A total of 11313 documents on foot and ankle were indexed in Scopus from 1980 to 2019. The annual number of documents showed a rapidly increasing pattern from 1980 to 2019, especially in the last decade (p = 0.000) (Fig.1). In total, 866 documents were published in 2019 in comparison to 13 documents published in 1980, indicating a 66.62-fold increase in documents from 1980 to 2019.

**Table-I:** Journal included in the current study.

Journal	Abbreviation	Impact factor
Foot & Ankle International	Foot Ankle Int	2.827
Foot and Ankle Surgery	Foot Ankle Surg	2.705
Journal of Foot Ankle Research	J Foot Ankle Res	2.303
Foot and Ankle Clinics	Foot Ankle Clin	1.653
Journal of Foot & Ankle Surgery	J Foot Ankle Surg	1.286

**Fig.2:**
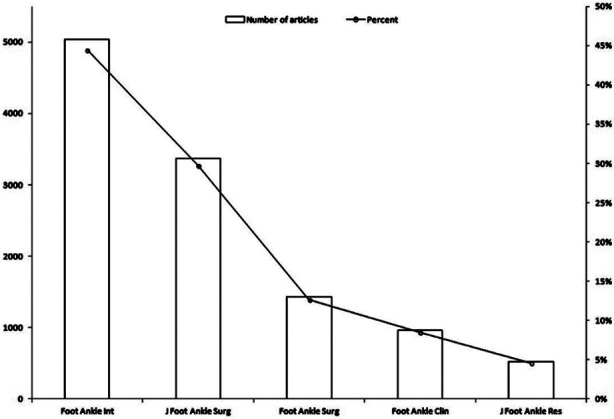
Documents published in five subspecialty foot and ankle journals.

The document ranking of the five foot and ankle journals. Foot & Ankle International ranked firs, with a total of 5032 documents (44.48%), followed by Journal of Foot & Ankle Surgery (29.74%), and Foot and Ankle Surgery (12.69%) (Fig.2).

The top 10 countries in terms of the number of documents on the foot and ankle are listed in Table-II. These countries contributed to 87.14% (9858/11313) of the total foot and ankle documents. The United States ranked first (5902; 52.17%), followed by the United Kingdom (1289; 11.39%), and Germany (410; 3.62%). The United States had the highest h-index (109), followed by the United Kingdom (48), and Australia (47).

**Table-II:** Top ten countries in foot and ankle research.

Rank	Country	N	%	H-index
1	United States	5902	52.17%	109
2	United Kingdom	1289	11.39%	48
3	Germany	410	3.62%	41
4	Australia	395	3.49%	47
5	South Korea	343	3.03%	29
6	Japan	326	2.88%	27
7	Switzerland	313	2.77%	40
8	Canada	309	2.73%	43
9	Italy	297	2.63%	31
10	Turkey	274	2.42%	25

The top 10 most productive institutions on foot and ankle. These institutions contributed 1138 documents (10.06%). Hospital for Special Surgery had the largest number of documents (212; 1.87%), followed by Union Memorial Hospital (155; 1.37%), and Duke University Medical Center (140; 1.24%). Union Memorial Hospital had the highest h-index (40), followed by Hospital for Special Surgery (37), and University of Southern California (31) (Table-III).

**Table-III:** Top ten institutions in foot and ankle research.

Rank	Institution	N	%	H-index
1	Hospital for Special Surgery	212	1.87%	37
2	Union Memorial Hospital	155	1.37%	40
3	Duke University Medical Center	140	1.24%	25
4	American Board of Podiatric Surgery	105	0.93%	26
5	University of Southern California	93	0.82%	31
6	La Trobe University	92	0.81%	25
7	Harvard Medical School	89	0.79%	19
8	VA Medical Center	86	0.76%	22
9	Amsterdam UMC	84	0.74%	24
10	UT Medical Banch at Galveston	82	0.72%	17

The top 10 productive authors on the foot and ankle are shown in Table-IV. These authors contributed 810 documents (7.16%). Myerson MS ranked first, with 141 publications (1.25%), followed by Schon LC (87; 0.77%), and Hyer CF (84; 0.74%). Myerson MS had the highest h-index (59), followed by Saltzman CL (54), and Hintermann B (51).

**Table-IV:** Top ten authors in foot and ankle research.

Rank	Author	N	%	H-index
1	Myerson MS	141	1.25%	59
2	Schon LC	87	0.77%	39
3	Hyer CF	84	0.74%	24
4	Deland JT	77	0.68%	35
5	Coughlin MJ	72	0.64%	45
6	Roukis TS	72	0.64%	25
7	Hintermann B	71	0.63%	51
8	Pinzur MS	69	0.61%	42
9	Saltzman CL	69	0.61%	54
10	Thordarson DB	68	0.60%	36

The top 10 cited documents are shown in Table-V. The number of citations in these documents ranged from 2717 to 259. Out of them, seven were published in Foot & Ankle International, and the other two were published in the Journal of Foot & Ankle Surgery.

**Table-V:** Top ten cited documents in foot and ankle research.

Rank	Author	Year	Title	Journal	Citations
1	Kitaoka HB	1994	Clinical rating systems for the ankle-hindfoot, midfoot, hallux, and lesser toes	Foot Ankle Int	2717
2	Wagner FW Jr	1981	The dysvascular foot: a system for diagnosis and treatment	Foot Ankle Int	611
3	Gerber JP	1988	Persistent disability associated with ankle sprains: a prospective examination of an athletic population	Foot Ankle Int	477
4	Frykberg RG	2006	Diabetic foot disorders: A clinical practice guideline (2006 revision)	J Foot Ankle Surg	425
5	Martin RL	2005	Evidence of validity for the Foot and Ankle Ability Measure (FAAM)	Foot Ankle Int	397
6	Saltzman CL	1995	The hindfoot alignment view		380
7	Roos EM	2001	Validation of the foot and ankle outcome score for ankle ligament reconstruction	Foot Ankle Int	337
8	Ibrahim T	2007	Reliability and validity of the subjective component of the American Orthopaedic Foot and Ankle Society clinical rating scales	J Foot Ankle Surg	307
9	Smith RW	1984	Hallux valgus assessment: report of research committee of American Orthopaedic Foot and Ankle Society	Foot Ankle Int	291
10	Lavery LA	1996	Classification of diabetic foot wounds	J Foot Ankle Surg	259

## DISCUSSION

In the present study, we sought to provide a scientometric overview of the research on the foot and ankle over the past four decades. Thus, we used the well-known Scopus database, which has been widely used in previous scientometric studies.^3^-^8^ Our study found that documents on foot and ankle increased rapidly over the past four decades, particularly in the past decade. The United States was the most active country, and had the highest quality of documents in terms of the h-index. Foot & Ankle International, with nearly half of all documents, was the most active journal. This is the first established journal in the five foot and ankle journal.

This might be attributed to the large number of documents in Foot & Ankle International.^4^,^8^,^23^ Moreover, Foot & Ankle International had the highest impact factor. These results suggest that the documents published in Foot & Ankle International in large quantities were also of high quality. Our findings demonstrate the significant impact of the Foot & Ankle International in the scientific community of foot and ankle. The current study found a 66.62-fold increase in the number of foot and ankle documents during the period 1980-2019. This may be an important turning point in the field of foot and ankle. The steep increase in the number of documents suggests notable progress in foot and ankle research in terms of scientometric theory.^3^-^8^

This indicates that global research on foot and ankle has thrived rapidly over the past four decades, particularly in the last 10 years. In addition, there were five foot and ankle journals in the Journal Citation Reports. The number of subspecialty journals may be higher than those of other subspecialties in orthopedics. These findings reflect the increasing concerns of scientific and medical committees regarding foot and ankle topics. The United States ranked first in terms of research output. This demonstrates the significant potential of the United States in scientific research. This might be due to the large number of high-level researchers and doctors, sufficient funding resources, and good medical policy.^4^,^6^,^8^,^24^

The United States was not only the most productive county for foot and ankle, but also had the highest h-index. This indicates that the United States made the greatest contribution to the quantity and quality of documents on foot and ankle research. The fact that research productivity differ in different countries may reveal different countries’ capabilities in science and technology.^3^,^4^,^6^,^8^,^25^-^28^ This scientometric analysis showed that more than 50% of documents published by authors from the United States, and nearly 90% of global foot and ankle documents were contributed by the top 10 countries. In addition, most of the top 10 countries were developed countries.

These results indicate that global research on foot and ankle was central. The phenomenon of a few countries contributing to a relatively large number of documents was also found in similar scientometric studies.^4^,^6^,^8^ This may be associated with the comprehensive potential of scientific research in each country, especially the development of education and economy.^4^,^6^,^8^,^23^ The majority of the top 10 institutions were located in the United States, which was led by the Hospital for Special Surgery. These institutions produce a great research output for the United States.

However, the Union Memorial Hospital had the highest h-index. This may indicate that the documents from the Union Memorial Hospital are of relatively higher quality than those from other institutions. In addition, the list of the top 10 authors showed authors with good records in foot and ankle research worldwide.^3^,^4^,^8^,^22^ These results show the great influence of these institutions and authors. Their contributions play an important role in the development of foot and ankle research.^4^,^8^ The top10 cited documents have been used to analyze hot spots and trends in foot and ankle research over the past four decades.

We found that the majority of documents discussed the rating system or outcome score. The top one documents had far more citations than any other documents. This may indicate that such a document is important for foot and ankle research, and has attracted increasing attention from other studies. Moreover, Foot & Ankle International published 7 of 10 top cited documents, which is considerably more than other foot and ankle journals. One possible reason for this might be that this journal was established earlier than the other journals. It is relatively easy for important documents published earlier to receive more citations.^4^,^8^,^23^ Another reason may be that this journal attracts more high-quality works than other journals.^3^,^6^,^8^

### Limitation:

The study has a few limitations, similar to those of previous scientometric analysis.^3^-^8^ Not all foot and ankle research has been reported in subspecialty foot and ankle journals. Other journals have also published foot and ankle documents. These journals could not be analyzed in the current study. Moreover, the number of journals included in this study was small. However, these journals present the main publications that have contributed to the field of foot and ankle surgery. Although Scopus is considered an excellent database for scientometric studies, false-negative and false-positive results remain unavoidable. Research activities on certain topics may have been under- or overestimated.

## CONCLUSION

Foot and ankle documents have dramatically increase over the past four decades, particularly in the last decade. The United States contributed the greatest number of documents, and had the highest quality of publications. The findings of this study could serve as baseline data for future comparative studies.

### Authors’ contributions:

**QL & YW:** Prepared this manuscript, are responsible and accountable for the accuracy and integrity of the work.

**QC & RC:** Collected and analyzed data, and significant revised this manuscript.
